# Chemical Characterization and Anti-Inflammatory Activity of *Eugenia involucrata* Essential Oil: Evidence from In Vitro and In Vivo Experimental Models

**DOI:** 10.3390/molecules31142477

**Published:** 2026-07-15

**Authors:** Kaique Gonçalves de Souza, Larissa Saviani Ribeiro, Vitor Guimarães Lourenço, Kevin Costa Miranda, Francisco Paiva Machado, Mariana Toledo Martins Pereira, Leandro Rocha, Vinicius D’Avila Bitencourt Pascoal, Aislan Cristina Rheder Fagundes Pascoal

**Affiliations:** 1 Research Laboratory of Natural Products and Bioactive Molecules, (Lab Nat—UFF) Nova Friburgo Health Institute, Fluminense Federal University—UFF, Nova Friburgo 28625-650, RJ, Brazil; kaiqueg@id.uff.br (K.G.d.S.); larissasaviani@id.uff.br (L.S.R.); vitorgl@id.uff.br (V.G.L.); viniciuspascoal@id.uff.br (V.D.B.P.); 2 Laboratory of Natural Products Technology, Department of Pharmaceutical Technology, School of Pharmacy, Fluminense Federal University, Niterói 24241-000, RJ, Brazil; kevincosta.cm@gmail.com (K.C.M.); fmachado@id.uff.br (F.P.M.); lean.machado@gmail.com (L.R.)

**Keywords:** *Eugenia involucrata*, essential oil, acute inflammation, TNF-α, IL-1β

## Abstract

Background: *Eugenia involucrata* DC. is a Brazilian native species of the Myrtaceae family traditionally used in folk medicine and known for its antimicrobial, antifungal, and antioxidant properties. However, its anti-inflammatory potential remains poorly investigated. Methods: This study characterized the essential oil from *E. involucrata* leaves and evaluated its anti-inflammatory activity using in vitro and in vivo models. The chemical composition was determined by gas chromatography–mass spectrometry (GC–MS). Anti-inflammatory activity was assessed in LPS-stimulated RAW 264.7 macrophages and in murine paw edema models induced by carrageenan, compound 48/80, bradykinin, and prostaglandin E_2_. Results: GC–MS analysis identified 23 compounds, representing 91.41% of the oil composition, with predominance of oxygenated sesquiterpenes. The major constituents were globulol (15.71%), α-selinene (13.14%), selin-11-en-4-α-ol (8.31%), cubeban-11-ol (8.04%), and β-elemene (6.78%). The essential oil showed no significant cytotoxicity below 100 micrograms/mL toward RAW 264.7 macrophages and significantly reduced LPS-induced TNF-α and IL-1β gene expression. In vivo, it markedly inhibited paw edema induced by carrageenan, compound 48/80, and bradykinin, but not by prostaglandin E_2_. Conclusions: These findings demonstrate that *E. involucrata* essential oil possesses significant anti-inflammatory and anti-edematogenic activity, likely through modulation of early inflammatory mediators involved in acute inflammation.

## 1. Introduction

Inflammation is a complex physiological process whose primary function is to eliminate harmful stimuli, such as pathogens and tissue injuries, as well as to remove necrotic cells and tissues resulting from these insults, thereby contributing to the maintenance of organismal homeostasis. This process involves a series of coordinated vascular and cellular alterations that characterize the five cardinal signs of inflammation: heat, redness, swelling, pain, and loss of function [1,2,3]. The inflammatory response may be classified as acute or chronic according to its duration and the organism’s ability to eliminate the injurious agent. Acute inflammation is characterized by a rapid response involving vasodilation, release of inflammatory mediators, and recruitment of leukocytes to the site of injury. When the inflammatory stimulus persists, the process may progress to chronic inflammation, which is marked by the continuous presence of inflammatory cells, sustained production of pro-inflammatory cytokines and reactive oxygen species, ultimately leading to tissue damage and remodeling [4]. Although inflammation represents an essential defense mechanism of the organism, dysregulated or persistent inflammation is associated with the development and progression of several diseases, including rhinitis, asthma, arthritis, neurodegenerative disorders, cancer, and inflammatory bowel diseases [1,4,5].

Several synthetic drugs are currently used in the treatment of inflammatory diseases; however, the prolonged use of these medications may be associated with significant adverse effects, raising concerns regarding therapeutic safety. Among the main classes employed are corticosteroids and nonsteroidal anti-inflammatory drugs (NSAIDs), which act by modulating different pathways of the inflammatory response. Nevertheless, long-term use of these agents may result in clinical complications, including Cushing’s syndrome, hypertension, hyperglycemia, gastrointestinal ulceration, and alterations in platelet function [6].

Therefore, there is a need to develop safe and effective therapeutic alternatives for the treatment of inflammatory diseases. In this context, natural products of plant origin stand out, as they have been used by several civilizations throughout human history and can treat up to 87% of diseases affecting humans due to their biological properties [7,8,9]. Numerous plant-derived compounds are recognized for their anti-inflammatory activity. Classical examples include acetylsalicylic acid, derived from salicylic acid isolated from Salix sp., colchicine, isolated from *Colchicum autumnale*, and the oil obtained from *Cordia verbenacea* DC. [10,11,12].

*Eugenia involucrata* DC. is a Brazilian species belonging to the family Myrtaceae and the genus Eugenia, popularly known as “cereja-do-mato”, “cerejeira-da-terra”, “cereja-do-rio-grande”, or “cereja-preta”. This species is used in culinary applications due to its edible fruits, in the restoration of degraded areas, and in traditional medicine for the treatment of diarrhea and dyspepsia [13,14,15]. Scientific studies conducted with this species suggest the presence of biological activities, including antifungal, antimicrobial, and antioxidant properties, thereby indicating its potential pharmacological relevance in the context of inflammation [16,17]. Therefore, the present study aims to evaluate the anti-inflammatory potential of the essential oil obtained from the leaves of *Eugenia involucrata* using in vitro and in vivo experimental models.

## 2. Results

The anti-inflammatory potential of *E. involucrata* essential oil (EIO) was investigated through a combination of chemical, in vitro, and in vivo approaches. Initially, the chemical composition of the essential oil was characterized by gas chromatography–mass spectrometry (GC–MS).

### 2.1. Phytochemical Profile Analysis

Phytochemical characterization by GC-MS allowed the identification of 23 compounds in the essential oil of *E. involucrata*, representing 91.41% of the essential oil. The analysis revealed a predominant fraction of oxygenated sesquiterpenes (47.47%), with the main components being globulol (15.71%), α—selinene (13.14%), selin-11-en-4-α-ol (8.31%), cubebin-11-ol (8.04%) and β-elemene (6.78%) (Table 1).

### 2.2. In Vitro Analysis of Anti-Inflammatory Effects

**Figure 1:**
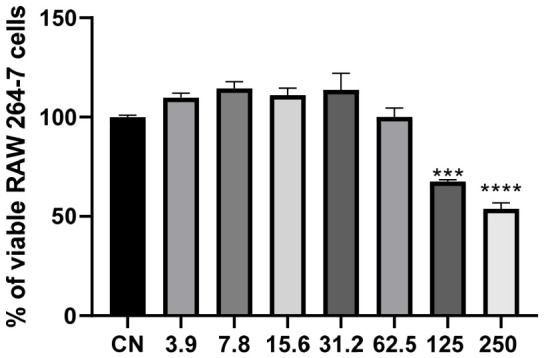
Analysis of cell viability in RAW 264.7 cells treated with the essential oil of *E. involucrata*, as determined by the MTT assay. Results were expressed as mean ± standard error of the mean (SEM). Differences between experimental groups and the control group were evaluated by one-way ANOVA followed by Dunnett’s multiple comparisons test. Values of *p* < 0.05 were considered statistically significant, *** = 0.0002, and **** < 0.0001.

The cytotoxicity of the sample was evaluated in RAW 264.7 macrophages using the MTT assay after exposure to increasing concentrations (3.9–250 µg/mL). As shown in Figure 1, treatment with the sample did not reduce cell viability at concentrations ranging from 3.9 to 62.5 µg/mL. On the contrary, cell viability remained above 100% at most concentrations, varying from approximately 104% to 118% relative to the untreated control, indicating the absence of cytotoxic effects and suggesting a possible increase in mitochondrial metabolic activity. However, at the highest concentrations tested (125 and 250 µg/mL), a concentration-dependent reduction in cell viability was observed, with mean viability values of approximately 67.5% and 53.8%, respectively. Despite this decrease, only the highest concentration resulted in cell viability below the 70% threshold commonly used to indicate cytotoxicity. These findings demonstrate that the sample exhibits good cytocompatibility toward RAW 264.7 macrophages at concentrations up to 250 µg/mL, supporting the use of lower concentrations in subsequent anti-inflammatory assays. Therefore, the IC_50_ value for EIO was determined to be 167.4 μg/mL.

Lipopolysaccharide (LPS) stimulation markedly increased the expression of the pro-inflammatory cytokines TNF-α and IL-1β in RAW 264.7 macrophages compared with untreated cells, confirming the successful establishment of the inflammatory model. As expected, activation of Toll-like receptor 4 (TLR4) by LPS triggers intracellular signaling pathways that culminate in NF-κB activation and the transcription of pro-inflammatory mediators, thereby promoting the initiation and amplification of the inflammatory response.

**Figure 2:**
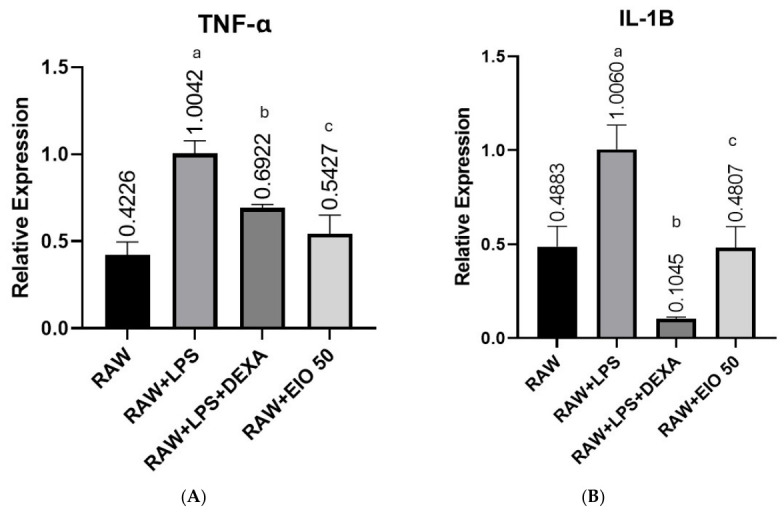

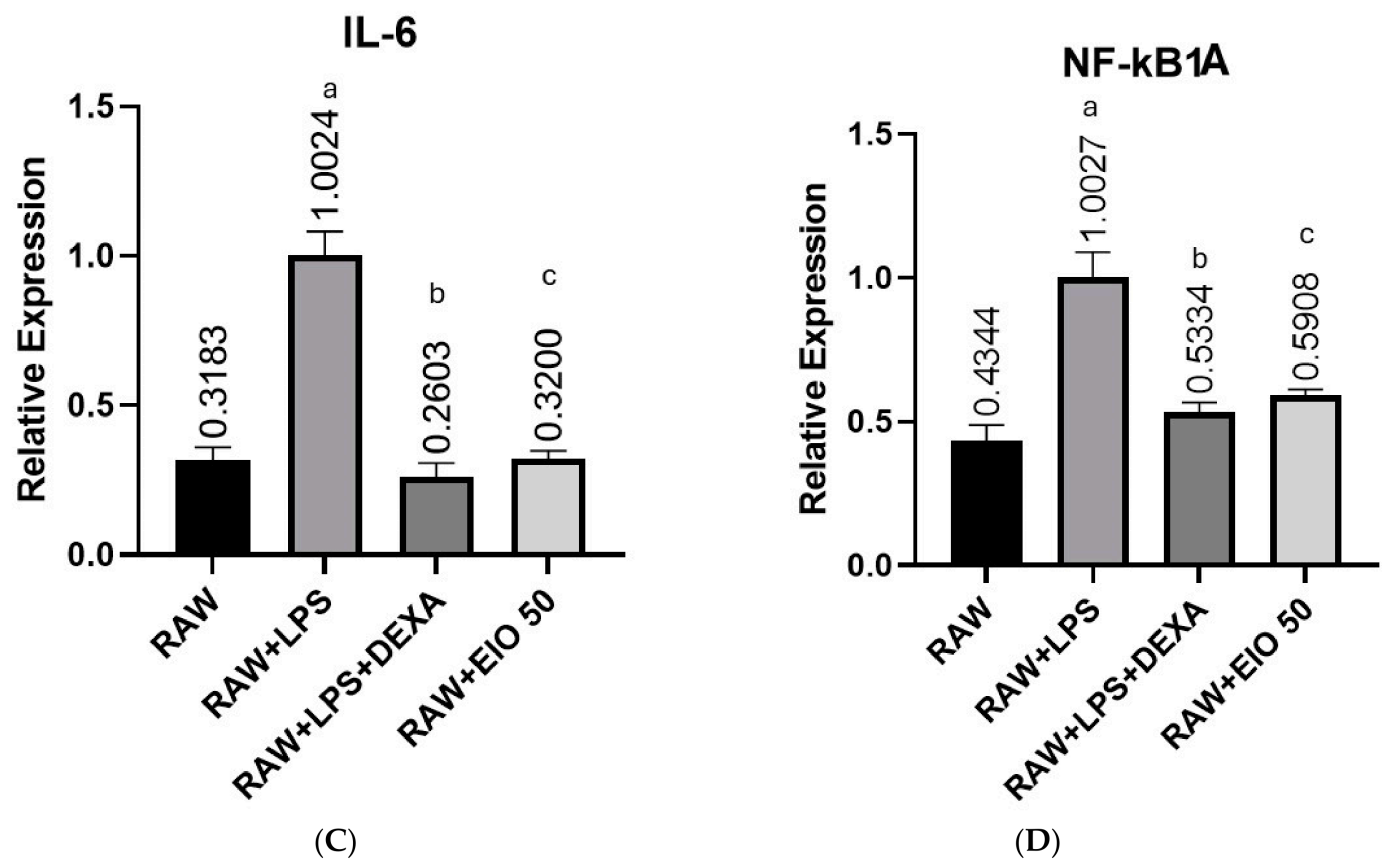
Effects of *E. involucrata* essential oil (EIO, 50 μg/mL) on TNF-α (**A**), IL-1β (**B**), Il-6 (**C**) and NFkB1a (**D**) gene expression in LPS-stimulated RAW 264.7 macrophages. Cells were treated with EIO or dexamethasone (DEXA, 1 μM) for 24 h in the presence of LPS. Gene expression was determined by RT-qPCR and normalized to the RAW group. Data are expressed as mean ± SEM. Different letters indicate statistically significant differences (*p* < 0.05): a differs from RAW and RAW + LPS + EIO; b differs from RAW + LPS; and c differs from RAW + LPS and RAW + LPS + EIO.

TNF-α gene expression was evaluated after 24 h of treatment with LPS in the absence or presence of *E. involucrata* essential oil (EIO, 50 μg/mL) or dexamethasone (1 μM), using LPS-stimulated cells as the inflammatory control (Figure 2A). LPS significantly increased TNF-α expression compared with untreated RAW 264.7 macrophages (mean difference = −0.5816; 95% CI: −0.7255 to −0.4377; *p* < 0.0001). Dexamethasone significantly attenuated this response, although expression levels remained higher than those observed in untreated cells (mean difference = −0.2696; 95% CI: −0.4135 to −0.1256; *p* = 0.0008). In contrast, treatment with EIO restored TNF-α expression to levels comparable with those of the untreated group, with no significant difference between these groups (mean difference = −0.1201; 95% CI: −0.2640 to 0.0239; *p* = 0.1085).

A similar pattern was observed for IL-1β (Figure 2B). LPS stimulation significantly increased gene expression compared with untreated macrophages (mean difference = −0.5177; 95% CI: −0.7303 to −0.3052; *p* < 0.0001). Dexamethasone markedly reduced IL-1β expression relative to the LPS-treated group (mean difference = 0.9016; 95% CI: 0.6891–1.114; *p* < 0.0001), although expression remained significantly lower than in untreated cells (mean difference = 0.3839; 95% CI: 0.1713–0.5964; *p* = 0.0008). Likewise, EIO significantly suppressed IL-1β expression compared with the LPS control (mean difference = 0.5254; 95% CI: 0.3128–0.7379; *p* < 0.0001), restoring expression to basal levels, as no significant difference was observed relative to untreated macrophages (mean difference = 0.0076; 95% CI: −0.2049 to 0.2202; *p* = 0.9995). Notably, IL-1β expression was significantly lower in the EIO-treated group than in dexamethasone-treated cells (mean difference = −0.3762; 95% CI: −0.5888 to −0.1637; *p* = 0.0010), suggesting a stronger inhibitory effect of the essential oil on this cytokine under experimental conditions.

Consistent with the previous findings, LPS significantly increased IL-6 gene expression compared with untreated macrophages (mean difference = 0.6840; *p* < 0.0001) (Figure 2C). Both dexamethasone and EIO effectively reduced this response, resulting in significantly lower IL-6 expression than that observed in the LPS-stimulated group (mean differences = 0.7421 and 0.6823, respectively; *p* < 0.0001 for both comparisons). Neither treatment differed significantly from the untreated control (dexamethasone: *p* = 0.4334; EIO: *p* > 0.9999), and no difference was detected between the EIO- and dexamethasone-treated groups (*p* = 0.4095), indicating that the essential oil exhibited an anti-inflammatory effect comparable to that of the reference drug.

LPS also significantly increased NF-κB1A gene expression compared with untreated RAW 264.7 cells (mean difference = 0.5683; *p* < 0.0001) (Figure 2D). Dexamethasone significantly reduced this increase (mean difference = 0.4693; *p* < 0.0001), restoring expression to levels that did not differ significantly from untreated cells (*p* = 0.1000). Similarly, EIO treatment significantly decreased NF-κB1A expression relative to the LPS-stimulated group (mean difference = 0.4119; *p* < 0.0001). Although expression remained slightly higher than that of untreated macrophages (mean difference = 0.1564; *p* = 0.0077), no significant difference was observed between EIO and dexamethasone (*p* = 0.4767), demonstrating that the essential oil effectively attenuated the LPS-induced inflammatory response.

### 2.3. In Vivo Analysis of Anti-Inflammatory Effects

**Figure 3:**
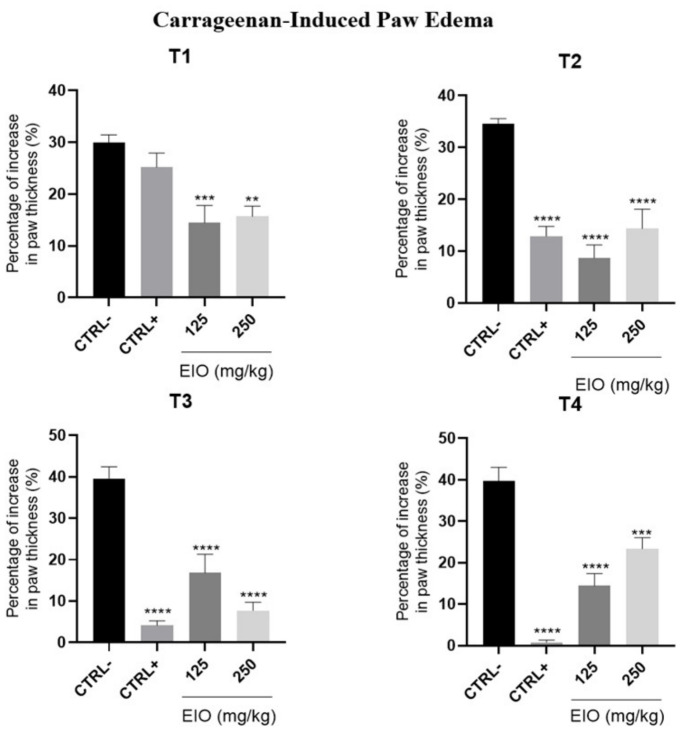
Anti-inflammatory effect of *E. involucrata* essential oil in the carrageenan-induced paw edema model. The effects of EIO at doses of 125 and 250 mg/kg, as well as indomethacin (10 mg/kg), were expressed as the percentage increase in paw thickness over time after edema induction. ** = *p* < 0.01, *** = *p* < 0.001, **** = *p* < 0.0001 compared with the saline-treated group.

The in vivo analyses using the mouse paw edema model allowed the evaluation of the anti-inflammatory and antiedematogenic effects of EIO against different phlogistic agents. In the carrageenan-induced paw edema model, both EIO doses showed statistically significant differences compared with the negative control at all experimental time points, indicating their ability to inhibit edema formation (Figure 3). The percentage inhibition of paw thickness ranged from 85.57% to 85.58% for EIO at 125 mg/kg and from 84.31% to 76.63% for EIO at 250 mg/kg. The positive control showed statistically significant differences starting from the second experimental time point, with inhibition percentages ranging from 74.29% to 99.17%.

These findings demonstrate that EIO possesses marked anti-inflammatory activity in the carrageenan-induced paw edema model, a widely used experimental model for evaluating acute inflammation. Since carrageenan-induced edema involves the sequential release of inflammatory mediators, including histamine, serotonin, bradykinin, and prostaglandins, the significant inhibition observed throughout the experimental period suggests that EIO may interfere with multiple stages of the inflammatory cascade. Notably, both tested doses exhibited inhibition rates comparable to or even higher than those observed for the reference drug at certain time points, indicating a potent antiedematogenic effect. The absence of a clear dose-dependent response may suggest that the lower dose was sufficient to achieve a near-maximal pharmacological effect under the experimental conditions. Collectively, these results reinforce the potential of EIO as a promising natural source of bioactive compounds with anti-inflammatory properties and support further investigations into its mechanisms of action and therapeutic applications.

**Figure 4:**
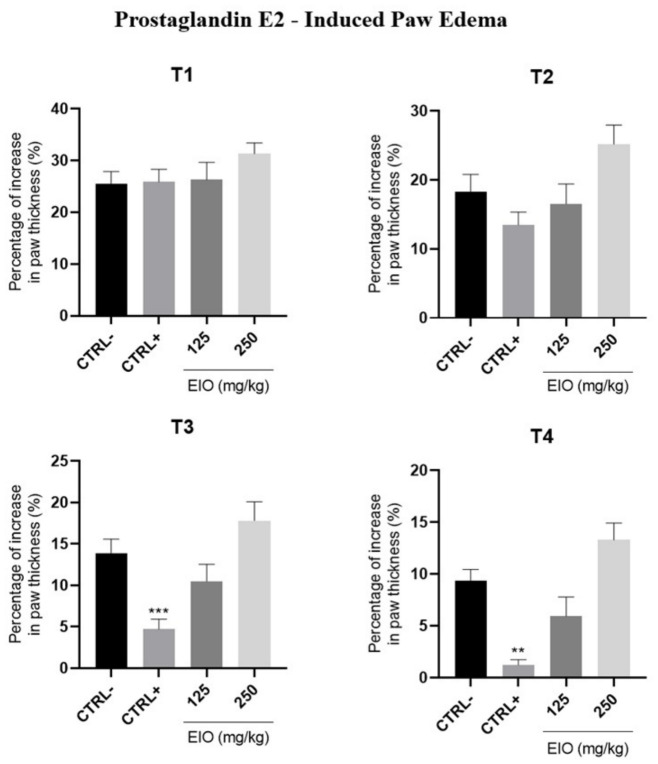
Anti-inflammatory effect of *E. involucrata* essential oil in the prostaglandin E2-induced paw edema model. The effects of EIO at doses of 125 and 250 mg/kg, as well as indomethacin (10 mg/kg), were expressed as the percentage increase in paw thickness over time after edema induction. Statistical significance was defined as *p* < 0.01 (**), *p* < 0.001 (***), compared with the saline-treated group.

In the prostaglandin E_2_-induced paw edema model, none of the experimental groups showed statistically significant differences compared with the negative control, indicating that EIO was unable to inhibit edema induced by this phlogistic agent under the conditions evaluated (Figure 4). In contrast, the positive control, indomethacin at 10 mg/kg, showed statistically significant effects at time points 3 and 4, inhibiting 95.29% and 98.79% of the induced edema, respectively.

In contrast to the results observed in the carrageenan-induced paw edema model, EIO did not significantly reduce edema formation in animals challenged with prostaglandin E_2_, suggesting that its anti-inflammatory activity is not primarily mediated through the inhibition of pathways downstream of prostaglandin action. Because prostaglandin E_2_ is a key mediator responsible for vasodilation and increased vascular permeability during inflammation, the lack of effect observed for EIO indicates that the essential oil may not directly antagonize prostaglandin receptors or interfere with the biological responses triggered by this mediator. The marked inhibition produced by indomethacin confirms the responsiveness of the experimental model and validates the assay conditions. Taken together, these findings suggest that the anti-inflammatory effects previously observed for EIO in the carrageenan-induced edema model may be associated with the modulation of upstream inflammatory mediators or signaling pathways involved in prostaglandin synthesis rather than the blockade of prostaglandin E_2_-induced responses themselves. Further mechanistic studies are necessary to clarify the specific targets involved in the anti-inflammatory action of EIO.

**Figure 5:**
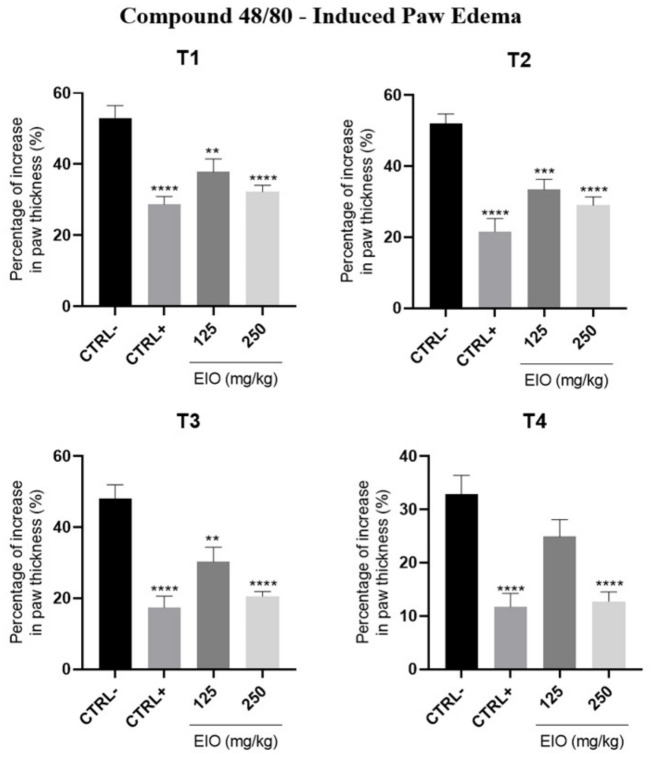
Anti-inflammatory effect of *E. involucrata* essential oil in the Compound 48/80-induced paw edema model. The effects of EIO at doses of 125 and 250 mg/kg, as well as cyproheptadine (4 mg/kg), were expressed as the percentage increase in paw thickness over time after edema induction. Statistical significance was defined as *p* < 0.01 (**), *p* < 0.001 (***), and *p* < 0.0001 (****) compared with the saline-treated group.

On the other hand, EIO was able to inhibit compound 48/80-induced paw edema from the first experimental measurement onward, showing statistically significant differences at the different time points evaluated (Figure 5). The EIO 125 mg/kg group showed edema inhibition percentages ranging from 62.19% to 75.14%, with significant differences compared with the negative control during the first three measurements. The EIO 250 mg/kg group showed statistically significant differences at all four time points, with inhibition percentages ranging from 67.74% to 82.26%. The positive control group was significant throughout the entire experiment and inhibited edema formation by 71.29% to 88.29%.

In the compound 48/80-induced paw edema model, EIO demonstrated a pronounced inhibitory effect on edema formation, indicating a potential modulatory action on mast cell-mediated inflammatory responses. Compound 48/80 is known to induce mast cell degranulation, leading to the rapid release of vasoactive mediators such as histamine and serotonin, which contribute to the development of acute edema. The significant reduction in paw swelling observed from the earliest time points suggests that EIO may interfere with the release or action of these mediators. Notably, the higher dose (250 mg/kg) produced significant inhibition throughout the entire experimental period and achieved inhibition rates comparable to those observed with the reference drug. These findings, together with the absence of activity in the prostaglandin E_2_-induced edema model, suggest that the anti-inflammatory effects of EIO may be more closely associated with the modulation of early inflammatory events, particularly those involving mast cell activation and the release of preformed inflammatory mediators. Therefore, the results provide important evidence that EIO exerts its anti-inflammatory activity, at least in part, through mechanisms related to the inhibition of mast cell-dependent pathways.

**Figure 6:**
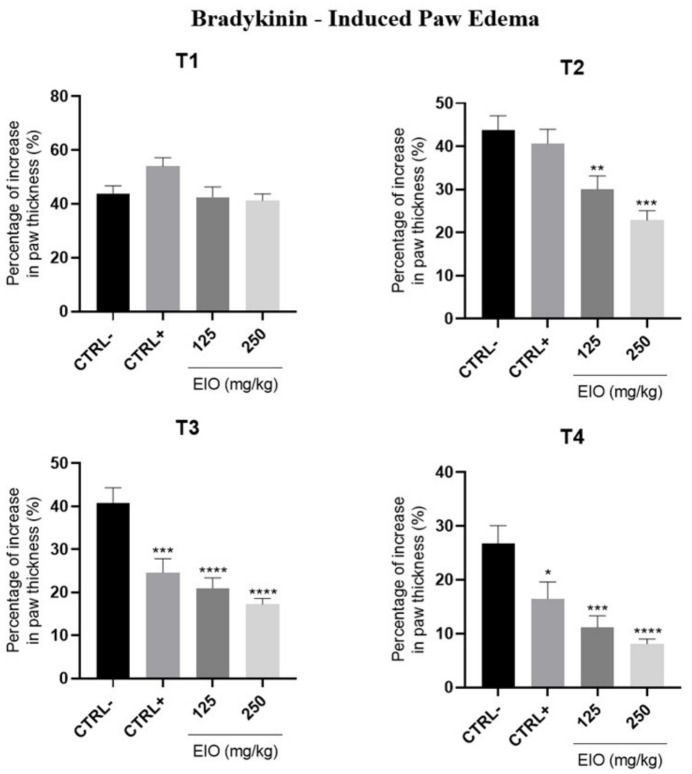
Anti-inflammatory effect of *E. involucrata* essential oil in the bradykinin-induced paw edema model. The effects of EIO at doses of 125 and 250 mg/kg, as well as dexamethasone (1 mg/kg), were expressed as the percentage increase in paw thickness over time after edema induction. Statistical significance was defined as *p* < 0.05 (*), *p* < 0.01 (**), *p* < 0.001 (***), and *p* < 0.0001 (****) compared with the saline-treated group.

Finally, in the bradykinin-induced paw edema model, EIO at doses of 125 mg/kg and 250 mg/kg showed statistically significant differences compared with the negative control starting from the second experimental time point, with edema inhibition percentages ranging from 57.52% to 88.87% and from 58.80% to 91.89%, respectively (Figure 6). The positive control, dexamethasone at 1 mg/kg, showed statistically significant differences during the last two measurements and was able to inhibit edema formation by 46.01% to 83.49%.

In the bradykinin-induced paw edema model, EIO significantly reduced edema formation at both tested doses, further supporting its anti-inflammatory potential. Bradykinin is a potent inflammatory mediator involved in vasodilation, increased vascular permeability, pain sensitization, and the stimulation of secondary mediators such as nitric oxide and prostaglandins. The significant inhibition observed from the second experimental time point onward suggests that EIO may interfere with bradykinin-mediated signaling pathways or with downstream inflammatory events triggered by this mediator. Notably, both doses produced inhibition percentages that were comparable to or even greater than those observed for dexamethasone at several time points, highlighting the strong anti-edematogenic activity of the essential oil.

When considered together with the results obtained in the compound 48/80 model, these findings indicate that EIO is particularly effective against inflammatory responses driven by early vasoactive mediators. The activity observed against bradykinin-induced edema, coupled with the lack of effect in the prostaglandin E_2_ model, suggests that EIO may act preferentially on specific inflammatory pathways involved in the initiation and amplification of acute inflammation rather than directly antagonizing prostaglandin-mediated responses.

## 3. Discussion

The present study demonstrated, for the first time, that the essential oil of *E. involucrata* (EIO) exhibits remarkable anti-inflammatory activity in both in vitro and in vivo models of acute inflammation. The essential oil was characterized by high levels of globulol (15.71%), α-selinene (13.14%), cubeban-11-ol (8.04%), and selin-11-en-4-α-ol (8.31%) as its major constituents. Previous phytochemical studies of *E. involucrata* leaf essential oil reported germacrene B, bicyclogermacrene, β-elemene, β-caryophyllene, globulol, and γ-elemene as the predominant compounds [17]. In contrast, compounds described as major constituents in those studies, such as β-caryophyllene and γ-elemene, were detected only at low concentrations or as minor constituents in the present work. These differences may be attributed to distinct edaphoclimatic conditions of the collection sites, as well as seasonal variations, cultivation practices, soil characteristics, water availability, plant developmental stage, and genetic factors, all of which are known to influence the biosynthesis of secondary metabolites and, consequently, the chemical composition of essential oils [19,20].

In the in vitro assays, EIO exhibited low cytotoxicity toward RAW 264.7 macrophages at concentrations below 125 μg/mL, allowing the evaluation of its pharmacological effects without compromising cell viability. At 50 μg/mL, EIO significantly reduced LPS-induced TNF-α expression, restoring its levels to those observed in non-stimulated cells. TNF-α is one of the earliest pro-inflammatory cytokines released by activated macrophages and plays a pivotal role in initiating and amplifying the inflammatory response through activation of the NF-κB and MAPK signaling pathways. Therefore, restoration of TNF-α expression to basal levels suggests that EIO interferes with early events of LPS-induced inflammatory signaling. Since TNF-α also promotes the production of downstream mediators, including IL-1β and IL-6, its inhibition may partly explain the concomitant reduction of these cytokines observed in the present study, indicating a broad modulatory effect of EIO on macrophage activation [21,22].

The anti-inflammatory activity observed for *E. involucrata* essential oil is unlikely to be attributed to a single constituent. Essential oils are complex mixtures of volatile compounds whose biological effects often result from synergistic or additive interactions among their constituents. The anti-inflammatory effect observed in the present study may therefore arise from the combined action of several sesquiterpenes identified in the essential oil, particularly β-caryophyllene, β-elemene, γ-elemene, spathulenol, viridiflorol, globulol, and palustrol. Notably, β-caryophyllene and spathulenol have been extensively shown to contribute to anti-inflammatory effects through the modulation of key inflammatory pathways, including inhibition of NF-κB activation, suppression of inducible nitric oxide synthase (iNOS) and cyclooxygenase-2 (COX-2) expression, reduction of pro-inflammatory cytokines such as TNF-α, IL-1β and IL-6, and attenuation of oxidative stress [23,24].

The anti-inflammatory activity of the isolated sesquiterpene selin-11-en-4α-ol has recently been demonstrated in LPS-stimulated RAW264.7 macrophages. Among its major constituents, selin-11-en-4α-ol, which accounted for 8.31% of the oil, has recently been reported as a promising anti-inflammatory compound in the same experimental model employed in the present study. Using LPS-stimulated RAW 264.7 macrophages, selin-11-en-4α-ol significantly inhibited the production of inflammatory mediators and chemokines, reduced reactive oxygen species generation, and suppressed the activation of both the MAPK and NF-κB signaling pathways, resulting in decreased inflammatory responses. Since NF-κB is a central transcription factor regulating the expression of TNF-α, IL-1β, and IL-6, the reduction of these cytokines observed in the present study is consistent with the mechanism previously described for selin-11-en-4α-ol. Although the biological activity of essential oils is generally attributed to synergistic interactions among multiple constituents, the relatively high abundance of this sesquiterpene suggests that it may substantially contribute to the anti-inflammatory effects of *E. involucrata* essential oil. This hypothesis is further supported by the concomitant downregulation of TNF-α, IL-1β, IL-6, and NF-κB1A observed in the present study, indicating modulation of key inflammatory signaling pathways [25]. Similar multimodal effects have also been reported for *E. pyriformis* essential oil, reinforcing that species of the genus Eugenia may share conserved anti-inflammatory mechanisms [26].

Although the pharmacological activities of several of the remaining sesquiterpenes remain poorly characterized, compounds such as globulol are frequently identified as major constituents of essential oils with anti-inflammatory activity. Therefore, the biological effects observed for EIO are likely to result from synergistic or additive interactions among multiple constituents rather than from the action of a single compound, a characteristic widely recognized for essential oils [27]. In this way, synergistic or additive interactions among these compounds may substantially contribute to the biological effects observed for *E. involucrata* essential oil.

In the carrageenan-induced paw edema model, *E. involucrata* essential oil (EIO) significantly inhibited edema formation throughout the entire experimental period. This model is widely used for the evaluation of anti-inflammatory agents because it involves a biphasic inflammatory response characterized initially by the release of histamine, serotonin, and bradykinin, followed by a later phase dependent on the production of prostaglandins, nitric oxide, and pro-inflammatory cytokines [28,29]. The ability of EIO to reduce edema from the earliest experimental time points and maintain this effect until the end of the evaluation suggests a broad action on multiple mediators involved in acute inflammation. Similar findings have been reported for sesquiterpene-rich essential oils, whose anti-inflammatory activity has been associated with the modulation of cytokine production and inflammatory lipid mediators [30].

Interestingly, EIO did not exhibit significant activity in the prostaglandin E_2_-induced edema model. This finding suggests that the oil does not act directly as an antagonist of prostaglandin receptors nor substantially interfere with the biological events triggered after prostaglandin receptor activation. Considering that prostaglandin E_2_ is a terminal mediator in the inflammatory cascade, the absence of activity in this model reinforces the hypothesis that the anti-inflammatory effects of EIO occur predominantly at earlier stages of the inflammatory response, possibly through the inhibition of the release or synthesis of upstream pro-inflammatory mediators. This pharmacological profile differs from that of classical nonsteroidal anti-inflammatory drugs but is consistent with the modulatory mechanisms frequently attributed to terpene-rich natural products [31].

The results obtained in the compound 48/80-induced paw edema model provide additional evidence supporting this mechanism of action. Compound 48/80 promotes intense mast cell degranulation, leading to the rapid release of histamine, serotonin, and other vasoactive mediators responsible for increased vascular permeability [32,33]. The marked inhibition of edema observed following EIO treatment suggests that its constituents may interfere with mast cell activation or attenuate the biological effects of the mediators released. Previous studies have demonstrated that several sesquiterpenes present in essential oils have been suggested to stabilize mast cells and reduce histamine release, thereby contributing to the attenuation of acute inflammatory responses [34,35].

In a complementary manner, EIO was also effective in the bradykinin-induced paw edema model. Bradykinin plays a central role in acute inflammation by promoting vasodilation, increasing vascular permeability, and inducing nociceptive sensitization, in addition to stimulating the production of nitric oxide, prostaglandins, and pro-inflammatory cytokines [36]. The ability of EIO to significantly reduce bradykinin-induced edema suggests that its constituents may interfere with signaling pathways associated with B1 and B2 receptors or modulate mediators produced secondary to receptor activation. The combined activity observed in the compound 48/80- and bradykinin-induced edema models, together with the lack of effect in the prostaglandin E_2_-induced model, points to a mechanism of action primarily related to the inhibition of early events in the acute inflammatory cascade.

Collectively, these findings suggest that EIO primarily targets upstream events of the inflammatory cascade. The simultaneous inhibition of NF-κB1A and pro-inflammatory cytokines, together with the absence of activity in the prostaglandin E_2_-induced edema model, indicates that the essential oil acts before prostaglandin-mediated signaling, possibly by modulating macrophage activation and the release of inflammatory mediators. This mechanism is consistent with the broad inhibitory profile observed in both the in vitro and in vivo assays. These findings expand the pharmacological knowledge of *E. involucrata* and reinforce the biotechnological potential of its essential oil as a promising source of bioactive compounds for the development of novel anti-inflammatory agents.

## 4. Materials and Methods

### 4.1. Plant Material and Essential Oil Extraction

Leaves of *E. involucrata* DC. were collected in Nova Friburgo, Rio de Janeiro State, Brazil, from cultivated specimens. The species was identified by a qualified botanist, and a voucher specimen was deposited in the Herbarium of the University of Campinas (UNICAMP) under accession number AEC 8890.

Fresh leaves (750 g) were subjected to hydrodistillation using a Clevenger-type apparatus for 4 h. The obtained essential oil was dried over anhydrous sodium sulfate and stored in amber vials at −20 °C until analysis.

### 4.2. GC–MS Analysis of the Essential Oil

The chemical composition of the essential oil was determined by gas chromatography coupled with mass spectrometry (GC–MS) using a GCMS-QP2010 (Shimadzu, Kyoto, Japan) equipped with an RTX-5MS fused silica capillary column (30 m × 0.25 mm i.d.; 0.25 μm film thickness; Restek Corporation, Bellefonte, PA, USA). The essential oil was diluted in dichloromethane (1000 ppm; ≥99.9% GC grade, Sigma-Aldrich, St. Louis, MO, USA), and 1.0 μL was injected in split mode (split ratio 1:40). Helium was used as the carrier gas at a constant flow rate of 1.0 mL min^−1^. The injector temperature was maintained at 260 °C, and the oven temperature was programmed from 60 °C to 290 °C at a heating rate of 3 °C min^−1^. Mass spectra were acquired by electron ionization (EI) at 70 eV with a scan rate of 1 scan s^−1^. Retention indices (RI) were calculated by interpolation using a homologous series of n-alkanes (C7–C40, Sigma-Aldrich, St. Louis, MO, USA) analyzed under the same chromatographic conditions. Compound identification was based on comparison of the calculated retention indices with literature data and comparison of the mass spectra and fragmentation patterns with those available in the NIST Mass Spectral Library and published literature [18].

### 4.3. Cell Culture

Murine macrophage RAW 264.7 cells were cultured in Dulbecco’s Modified Eagle Medium (DMEM) supplemented with 10% fetal bovine serum (FBS) and 1% penicillin–streptomycin. Cells were maintained at 37 °C in a humidified atmosphere containing 5% CO_2_.

### 4.4. Cell Viability Assay

Cell viability was assessed using the MTT assay. RAW 264.7 cells were seeded in 96-well plates (3.5 × 10^4^ cells/well) and incubated for 24 h. Cells were then treated with different concentrations of the essential oil for 48 h. Subsequently, MTT solution (5 mg/mL) was added and incubated for 4 h. The resulting formazan crystals were dissolved in DMSO, and absorbance was measured at 570 nm using a microplate reader (Epoch, BioTek Instruments, Winooski, VT, USA). Cell viability was expressed as a percentage relative to untreated control cells [27].

### 4.5. Quantitative Real-Time PCR (RT-qPCR)

RAW 264.7 cells were stimulated with lipopolysaccharide (LPS, 1 μg/mL) in the presence or absence of *E. involucrata* essential oil. Total RNA was extracted using TRIzol reagent (Invitrogen, Carlsbad, CA, USA), and cDNA was synthesized from 2 μg of RNA using the High-Capacity cDNA Reverse Transcription Kit (Thermo Fisher Scientific, Waltham, MA, USA).

Gene expressions of TNF-α, IL-1β, IL-6 and NF-kB1a was determined by quantitative real-time PCR using Power SYBR Green PCR Master Mix (Thermo Fisher Scientific, USA). GAPDH was used as the reference gene. Relative gene expression levels were calculated using the 2^−ΔΔCt^ method [37].

Primer sequences were as follows:

GAPDH: Forward 5′-GTCTTC ACTACCATGGAGAAGG-3′; Reverse 5′-TCATGGATGACCTTGGCCAG-3′

TNF-α: Forward 5′-AGGGGAAATGAGAGACGCAA-3′; Reverse 5′-TTCCCCATCTCTTGCCACAT-3′

IL-1β: Forward 5′-TGCAGAGTTCCCCAACTGGTACATC-3′; Reverse 5′-GTGCTGCCTAATGTCCCCTTGAATC-3′

NFkB1a: Forward 5′-AGAACAACCTGCAGCAGACT-3′; Reverse 5′-TAGACACGTGTGGCCATTG-3′

Il-6: Forward 5′-GATTCAATGAGGAGACTTGCC-3′; Reverse 5′-TGTTCTGGAGGTACTCTAGGTA-3′

### 4.6. Animals

Adult female Swiss mice (25–30 g) were obtained from the Laboratory Animal Center of Fluminense Federal University (NAL-UFF). Animals were housed under controlled temperature (22 ± 2 °C), relative humidity (45–55%), and a 12 h light/dark cycle, with free access to food and water. All experimental procedures were approved by the Ethics Committee for Animal Use of Fluminense Federal University under protocol number 1791100425.

### 4.7. Carrageenan-Induced Paw Edema

Anti-inflammatory activity was evaluated using the carrageenan-induced paw edema model [38]. Mice were randomly allocated into four groups (*n* = 8): saline (negative control), indomethacin (10 mg/kg, positive control), and *E. involucrata* essential oil at 125 or 250 mg/kg. Treatments were administered orally 30 min before intraplantar injection of carrageenan (30 μg/paw).

Paw thickness was measured before and 1, 2, 3, and 4 h after carrageenan administration using a digital caliper. Results were expressed as percentage increase relative to baseline values.

### 4.8. Paw Edema Induced by Prostaglandin E_2_, Compound 48/80, and Bradykinin

The anti-inflammatory activity of the essential oil was further evaluated using paw edema induced by prostaglandin E_2_ (30 μg/paw), compound 48/80 (10 μg/paw), or bradykinin (50 μg/paw). Animals were pretreated orally with saline, essential oil (125 or 250 mg/kg), or the respective reference drugs: indomethacin (10 mg/kg), cyproheptadine (4 mg/kg), and dexamethasone (1 mg/kg).

Thirty minutes after treatment, inflammatory agents were administered intraplantarly into the right hind paw. Paw thickness was measured before and at 15, 30, 60, and 90 min after induction. Results were expressed as percentage edema relative to baseline values [39].

### 4.9. Statistical Analysis

Data are presented as mean ± SEM. Statistical analyses were performed using one-way ANOVA followed by Dunnett’s multiple comparisons test. Differences were considered statistically significant when *p* < 0.05.

## 5. Conclusions

This study demonstrated that the essential oil obtained from the leaves of *E. involucrata* possesses a sesquiterpene-rich chemical composition, with globulol, α-selinene, cubeban-11-ol, and selin-11-en-4-α-ol identified as the major constituents. The essential oil exhibited low cytotoxicity at pharmacologically relevant concentrations and significantly suppressed the LPS-induced expression of the pro-inflammatory cytokines TNF-α, IL-1β, and IL-6 in RAW 264.7 macrophages. In addition, treatment with the essential oil significantly reduced NFKBIA gene expression, reaching levels comparable to those observed with dexamethasone, indicating modulation of the NF-κB signaling pathway.

The in vivo experiments further demonstrated that the essential oil significantly inhibited edema induced by carrageenan, compound 48/80, and bradykinin, whereas no inhibitory effect was observed against prostaglandin E_2_-induced edema. Together, these findings indicate that the anti-inflammatory activity of *E. involucrata* essential oil is primarily associated with the modulation of early inflammatory signaling events, including NF-κB-related pathways and the production of pro-inflammatory cytokines, as well as the actions of vasoactive mediators involved in the initial phase of acute inflammation. In contrast, the lack of effect on prostaglandin E_2_-induced edema suggests that prostaglandin-mediated signaling is unlikely to represent its primary pharmacological target.

Overall, the present study supports a mechanistic model in which *E. involucrata* essential oil predominantly acts by regulating early inflammatory responses through modulation of NF-κB-associated signaling and downstream cytokine production, rather than directly interfering with prostaglandin-dependent pathways. These findings expand the current pharmacological knowledge of *E. involucrata* and support its essential oil as a promising source of bioactive compounds for the development of anti-inflammatory agents. Future studies should identify the constituents responsible for these effects and further investigate the intracellular molecular targets involved in NF-κB signaling and other upstream regulatory pathways.

## Figures and Tables

**Table 1:** Chemical characterization of *E. involucrata* essential oil from leaves by GC-MS.

	RT (min)	AI_rep_	AI_Lit_	Substances	Relative Abundance (%)
1	22.495	1392	1389	**β-elemene**	**6.78**
2	23.592	1419	1417	β-caryophyllene	2.28
3	24.185	1433	1434	γ-elemene	0.98
4	24.397	1439	1439	Aromadendrene	1.66
5	25.277	1460	1464	9-epi-(E)-caryophyllene	2.16
6	25.882	1475	1476	β-chamigrene	5.82
7	26.313	1486	1489	β-selinene	5.37
8	26.681	1495	1498	**α-selinene**	**13.14**
9	26.900	1501	1500	α-muurolene	0.80
10	28.236	1535	1528	Zonarene	1.72
11	28.495	1542	1545	Selina-3,7(11)-diene	0.93
12	28.910	1553	-	n.i.	1.13
13	29.076	1557	1559	Germacrene B	2.30
14	29.482	1567	1567	Palustrol	3.50
15	29.894	1578	1577	Spathulenol	1.10
16	30.142	1585	1590	**Globulol**	**15.71**
17	30.447	1592	1592	Viridiflorol	4.17
18	30.526	1594	1595	**Cubeban-11-ol**	**8.04**
19	30.833	1603	1600	Rosifoliol	3.84
20	31.258	1614	-	n.i.	2.10
21	32.346	1644	1640	epi-α-muurolol	1.22
22	32.805	1656	1658	**Selin-11-en-4-α-ol**	**8.31**
23	34.310	1697	1700	Eudesm-7(11)-en-4-ol	1.58
24	35.661	1735	-	n.i.	4.50
25	40.445	1875	-	n.i.	0.85
Total identified					91.41
Not identified (n.i.)					8.58
Sesquiterpene hydrocarbons					43.94
Oxygenated sesquiterpenes					47.47

RT, retention time; AI_Lit_, arithmetic index from literature (Adams, 2017 [18]); AI_rep_, arithmetic index reported; n.i., not identified. Compounds in bold are the major ones.

## Data Availability

The original contributions presented in this study are included in the article. Further inquiries can be directed to the corresponding author.
